# Correlation between Relaxometry and Diffusion Tensor Imaging in the Globus Pallidus of Huntington’s Disease Patients

**DOI:** 10.1371/journal.pone.0118907

**Published:** 2015-03-17

**Authors:** Michael Syka, Jiří Keller, Jiří Klempíř, Aaron M. Rulseh, Jan Roth, Robert Jech, Ivan Vorisek, Josef Vymazal

**Affiliations:** 1 Department of Radiology, Na Homolce Hospital, Prague, Czech Republic; 2 International Clinical Research Center, St. Anne´s University Hospital, Brno, Czech Republic; 3 Department of Neurology, 3rd Faculty of Medicine, Charles University in Prague, Prague, Czech Republic; 4 Department of Neurology and Center of Clinical Neuroscience, 1st Faculty of Medicine and General University Hospital in Prague, Charles University in Prague, Prague, Czech Republic; 5 Institute of Anatomy, 1st Faculty of Medicine, Charles University in Prague, Prague, Czech Republic; 6 Department of Radiology, 1st Faculty of Medicine and General University Hospital in Prague, Charles University in Prague, Prague, Czech Republic; 7 Institute of Experimental Medicine, Academy of Sciences of the Czech Republic, v.v.i., Prague, Czech Republic; University of Minnesota, UNITED STATES

## Abstract

Huntington's disease (HD) is an inherited neurodegenerative disorder with progressive impairment of motor, behavioral and cognitive functions. The clinical features of HD are closely related to the degeneration of the basal ganglia, predominantly the striatum. The main striatal output structure, the globus pallidus, strongly accumulates metalloprotein-bound iron, which was recently shown to influence the diffusion tensor scalar values. To test the hypothesis that this effect dominates in the iron-rich basal ganglia of HD patients, we examined the globus pallidus using DTI and T2 relaxometry sequences. Quantitative magnetic resonance (MR), clinical and genetic data (number of CAG repeats) were obtained from 14 HD patients. MR parameters such as the T2 relaxation rate (RR), fractional anisotropy (FA) and mean diffusivity (MD) were analysed. A positive correlation was found between RR and FA (R2=0.84), between CAG and RR (R2=0.59) and between CAG and FA (R2=0.44). A negative correlation was observed between RR and MD (R2=0.66). A trend towards correlation between CAG and MD was noted. No correlation between MR and clinical parameters was found. Our results indicate that especially magnetic resonance FA measurements in the globus pallidus of HD patients may be strongly affected by metalloprotein-bound iron accumulation.

## Introduction

Huntington's disease (HD) is an incurable hereditary neurodegenerative disorder with severe impairment of motor and cognitive functions and behavioral abnormalities. HD is caused by a mutation of the *IT 15* gene on the short arm of chromosome 4 [1,2]. This mutation is based on the expansion of the cytosine-adenine-guanine (CAG) triplet [3], and the gene product is called huntingtin [4]. In its adult form, most HD patients become symptomatic during the fourth or fifth decade of life. The clinical presentation may start with psychiatric symptoms such as irritability, depression or compulsive behavior; however, neurological symptomatology with choreatic dyskinesias and cognitive decline later develop in almost all patients. The progressive motor and cognitive deterioration leads to immobility, dementia and premature death. The clinical features of HD are closely related to the degeneration of the striatum and its connections with the frontal lobes [5]. The principal structural change, which starts early in the disease course, is a severe atrophy of the striatum with selective degeneration of the GABAergic medium spiny neurons, which mainly project to the globus pallidus (pallidum). In the advanced stages of HD the loss of neurons becomes widespread, also involving the cortex and cerebellum [6,7,8]. The clinical assessment of symptoms in HD is mostly made using the Unified Huntington's Disease Rating Scale (UHDRS), which rates four aspects of clinical performance: motor function, cognitive function, behavioral abnormalities and functional capacity [9].

Standard magnetic resonance (MR) imaging studies of HD show atrophy of the head of the caudate, leading to dilatation of the frontal horns of the lateral ventricles; general brain atrophy usually follows. Recently, a number of studies on HD patients have used quantitative magnetic resonance diffusion-weighted imaging (DWI), especially diffusion tensor imaging (DTI) [10,11,12,13,14]. DWI and DTI describe the molecular aspects of water diffusion, which may consequently provide some information about tissue microstructure. Two indices frequently reported, mean diffusivity (MD) and fractional anisotropy (FA), can be derived from the diffusion tensor [15]. MD is a measure of the magnitude of water diffusion in the tissue, and is affected by intra- and extracellular volume, cell permeability and extracellular space content, while FA reflects the degree of anisotropic diffusion in a given voxel and thus may be affected by tissue integrity, fiber composition or the selective loss of white matter bundles. Generally, DTI studies usually focus on white matter changes, but they also show great promise for assessing gray matter processes [11,12,13,14,16], despite the fact that gray matter is relatively isotropic on a voxel basis.

Another quantitative MR technique employed in studies of HD patients, T2 relaxometry, is used for analyzing the T2 relaxation time or its reciprocal value, the T2 relaxation rate (RR). T2 relaxation time is thought to be considerably affected by the presence of iron in the metalloprotein ferritin [17,18,19,20] and in its water-insoluble degradation product hemosiderin. Under physiological conditions the highest amount of iron per tissue weight is found in the pallidum, which increases with aging [21,22,23,24]. It has also been demonstrated that changes in the level and form of brain iron varies according to different neurological diseases [25,26] and that these changes may play an important role in the pathogenesis of neurodegenerative diseases [27,28]. In HD, alterations of the T2 relaxation time were demonstrated in the basal ganglia (BG) and white matter [27,29,30], particularly in the pallidum [30]. However, while many studies suggest different pathological mechanisms [31,32,33], the role of iron in the pathogenesis of HD has not yet been clarified.

Recently it has been shown that the presence of higher levels of metalloprotein-bound iron in the gray matter affects DTI scalar values such as fractional anisotropy (FA) and mean diffusivity (MD) [34,35]. Moreover, certain DTI measurements in the extrapyramidal nuclei correlate with T2 relaxation times [34,35,36], above all in the pallidum [35]. Correlations have been observed between metalloprotein-bound iron concentration, RR and signal-to-noise ratio (SNR) [35]. With increasing RR (decreasing SNR) an artifactual increase of FA and decrease of MD were noted both *in vitro* and *in vivo* [35].

Therefore, we aimed to study whether the same correlation among FA, MD and RR exists in the pallidum of HD patients, the area in which we expected the correlation to be most pronounced. Furthermore, we investigated whether FA and MD values correlate with the genetic load or certain clinical parameters of the disease. To test our hypothesis, we examined the pallidum of HD patients using DTI and T2 relaxometry. A similar methodology using the same magnetic field strength was adopted from our recently published study dealing with the effect of metalloprotein-bound iron on DTI in healthy subjects [35].

## Materials and Methods

### Ethics Statement

The study was approved by the local ethics committee of Na Homolce Hospital, and was performed in accordance to the ethical standards prescribed in the 1964 Helsinki Declaration. Furthermore all patients and control subjects provided signed, informed consent. Details that might disclose the identity of the subjects under study have been omitted.

### Patients and control subjects

Eighteen HD patients with a known age of onset of overt motor manifestations were consecutively enrolled in the study. General exclusion criteria included low compliance, severe disability and involuntary movements that would interfere with the MR procedure. Nevertheless, four of the examined patients were later excluded due to low quality MR images (motion artefacts or head tilt). From the remaining group of patients, only the right pallidum could be examined in one patient (due to head tilt). Seven patients from the further analysed group were treated with neuroleptics during the course of the illness. The average age of the group of 14 patients (7 females and 7 males) was 49.5 ± 12 (SD) years with a range of 28–68 years; median 54 years.

In all patients a neurological and genetic examination was performed. The severity of clinical motor impairment was assessed using a motor subscale of the UHDRS. This motor subscale consists of 15 items which rate individual motor symptoms of HD, ranging from 0 (symptom absent) to 4 (highest severity), and their sum indicates the total UHDRS motor score. When describing the functional disability and disease stage of HD patients, a Total Functional Capacity (TFC) score, another subscale of the UHDRS, was used. The TFC score rates 5 general patient skills. Specifically, occupation, finances, domestic chores, daily life activities and level of care, where a lower total score corresponds to a higher severity of HD. The number of CAG triplet repeats was determined using a deoxyribonucleic acid (DNA) analysis of leukocytes taken from the peripheral blood and amplification by the polymerase chain reaction (PCR) [37]. The disease burden score (DBS) was calculated by multiplying the number of CAG triplet repeats beyond 35.5 and the age of the patient at the time of examination [38].

Fourteen age-matched healthy control subjects (7 females and 7 males) were scanned using the same MR protocol. The results of one control subject were excluded from further analysis due to the poor quality of MR images. The average age was 47.6 ± 12.4 (SD) years with a range of 30–69 years. All control subjects were free of neurological and psychiatric diseases.

### MR protocol

All imaging was performed at 1.5T with a Siemens Symphony syngo scanner (Siemens Magnetom; Erlangen, Germany). Structural imaging was performed with a double-echo proton density (PD) and T2-weighted turbo spin echo (TSE) sequence covering the whole brain with 5 mm thick slices. These images were also used to exclude other visible pathology such as brain neoplasm.

Diffusion data was acquired by an SE-EPI work in progress (WIP) sequence with the following parameters: 30 non-collinear gradient directions, b = 1100 s/mm^**2**^, five b0 images, 70 transversal slices, TR = 7700 ms, TE = 102 ms, voxel size 2.5 x 2.5 x 2.5 mm, FOV 240 mm, NEX 1, acceleration factor 2.

**Fig 1:**
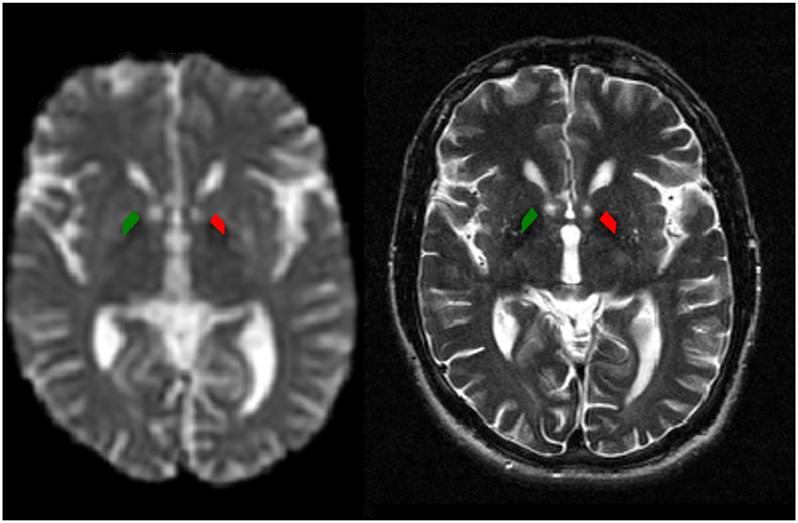
ROIs definition. The placement of the ROI masks on the CPMG sequence (right) and their projection on the co-registered MD map in an HD patient (left).

For T2 relaxometry the scanning protocol included a single slice Carr-Purcell-Meiboom-Gill-type (CPMG) multiple echo sequence, positioned on the axial plane approximately parallel to the plane passing through the anterior/posterior commissures to optimally cover the basal ganglia (Fig. 1). The parameters of the CPMG sequence were: 32 interecho times of 12.5, 25.0, 37.5 to 400.0 ms, TR = 3000 ms, FOV 230 mm, voxel size: 0.9 x 0.9 x 4 mm.

### DTI and relaxometry data evaluation

The diffusion-weighted images were processed off-line using FSL 4.1 [39], which included eddy-current correction and motion correction. Brain extraction, using a brain extraction tool (BET) [40], was performed in the next step.

DTIFit (a FSL program that fits a diffusion tensor model at each voxel of diffusion images) was then used to calculate the scalar invariants of the tensor. The generated 3D-images included MD, FA, and a raw T2-signal image. The raw T2-signal image (with no diffusion weighting) generated by DTIFit had the same distortions as the DTI maps it produced. CPMG position was copied from the slice position of one of the slices of the whole-head T2 sequence. A single-slice CPMG image of the corresponding TE was used for 2D co-registration of CPMG to T2. A T2 image derived from the diffusion data was then linearly co-registered to the whole-head T2, and the transformation was applied to the FA/MD maps. A single slice (corresponding to CPMG) was then extracted from the resulting volume. This approach produced four single co-registered slices (FA, MD, T2 and CPMG). In FSLView, masks of the bilateral regions of interest (ROIs) in the pallidum were first manually created on the CPMG data and then projected onto the co-registered FA/MD maps. One example is shown in Fig. 1. In the patient group, where due to atrophy a relatively small pallidum was present, ROIs were placed by two independent radiologists (MS and JV). In the healthy control group, where a relatively larger pallidum could be easily observed, ROIs were placed only by one radiologist (MS). The average signal data for each echo time was stored, generating 32 intensity values for each mask. T2 relaxation times were calculated by curve-fitting with a single exponential decay function using a free baseline in GraphPad Prism 5 (GraphPad Software Inc., La Jolla, CA, USA).

### Statistical analysis

Statistical analyses was performed using R—a language and environment for statistical computing (R foundation for statistical computing, Vienna, Austria, 2010)—and GraphPad InStat version 3.10 (GraphPad Software Inc., La Jolla, CA, USA). The results from both independent radiologists were first averaged to reduce the systematic error of both readers, and these averages were then used in further analyses. As no differences in RR, FA or MD were found between the left and right pallidum, the results from both sides were averaged. A linear model was used to correlate RR, FA and MD with CAG, UHDRS, TFC, patient age, disease onset, DBS and disease duration. The Welch two sample t-test was used for intergroup comparison as normality was not determined. To correct for multiple comparisons the Benjamini and Hochberg false discovery rate (FDR) was controlled to maintain an alpha value of 0.05. The correlation is considered to be significant where P<0.05, at the same time as P<FDR.

## Results

The clinical and genetic data obtained from HD patients are listed in Table 1. The relaxometry data—RR—and the diffusion data—FA and MD—obtained from the left and right pallidum of HD patients and healthy controls with the corresponding standard deviations (SD) are listed in Table 2. The P and R2 values for the correlations of the data from HD patients, with the FDR correction for multiple comparisons, are listed in Table 3.

**Table 1:** The clinical and genetic data obtained from HD patients.

Patient number	TFC value	UHDRS value	CAG count	HD duration (years)	Age (years)	Sex
1	8	30	48	3	36	M
2	2	80	44	8	49	F
3	8	15	42	2	54	M
4	1	24	43	4	56	F
5	7	10	39	10	58	M
6	10	9	44	2	28	M
7	7	15	40	7	68	M
8	12	23	54	2	31	F
9	3	13	44	8	41	F
10	13	24	42	3	58	M
11	5	29	42	6	61	F
12	3	22	45	12	54	F
13	4	24	43	9	44	F
14	7	20	43	6	56	M

**Table 2:** The relaxometry data—mean RR (s^**-1**^) and the diffusion data—FA (without units) and MD (mm^**2**^s^**-1**^) obtained from the pallidum (GP) of HD patients and healthy controls with the corresponding standard deviations (SD).

LEFT GP	RR	RR (SD)	FA	FA (SD)	MD
patients—1. observer	17.57	2.99	0.46	0.13	0.000611
patients—2. observer	16.41	2.10	0.45	0.17	0.000595
patients—average of observers	16.85	2.52	0.44	0.15	0.000609
healthy controls	15.04	0.57	0.37	0.07	0.000647

RIGHT GP	RR	RR (SD)	FA	FA (SD)	MD
patients—1. observer	16.79	2.54	0.42	0.10	0.000659
patients—2. observer	15.52	1.52	0.47	0.15	0.000598
patients—average of observers	16.05	1.98	0.44	0.12	0.000632
healthy controls	14.92	0.64	0.36	0.08	0.000644

LEFT+RIGHT GP	RR	RR (SD)	FA	FA (SD)	MD
patients—average of observers	16.45	0.53	0.44	0.03	0.000616
healthy controls	14.98	0.12	0.36	0.03	0.000646

**Table 3:** Statistical data obtained for the correlations of the results from HD patients—P values with the FDR correction for multiple comparisons and R2 values.

Correlation	P value	FDR	R2 value	Conclusion
**FA x MD**	**<0.001**	**0.002**	**0.85**	**significant**
**RR x FA**	**<0.001**	**0.005**	**0.84**	**significant**
**RR x MD**	**<0.001**	**0.007**	**0.66**	**significant**
**CAG x RR**	**0.001**	**0.010**	**0.59**	**significant**
**CAG x FA**	**0.010**	**0.012**	**0.44**	**significant**
**CAG x MD**	**0.037**	**0.014**	**0.31**	**trend**
FA x UHDRS	0.052	0.017	0.27	not significant
FA x TFC	0.276	0.04	0.06	not significant
FA x Age	0.155	0.031	0.16	not significant
FA x Onset	0.234	0.038	0.11	not significant
FA x Duration	0.297	0.043	0.09	not significant
MD x UHDRS	0.138	0.029	0.17	not significant
MD x TFC	0.097	0.021	0.20	not significant
MD x Age	0.137	0.024	0.17	not significant
MD x Onset	0.309	0.045	0.08	not significant
MD x Duration	0.056	0.019	0.27	not significant
RR x UHDRS	0.170	0.033	0.15	not significant
RR x TFC	0.373	0.050	0.02	not significant
RR x Age	0.138	0.026	0.17	not significant
RR x Onset	0.212	0.036	0.13	not significant
RR x Duration	0.319	0.048	0.08	not significant

The correlation is considered to be significant if P<0.05 and at the same time P<FDR.

### Correlation of relaxometry, diffusion, genetic and clinical results from HD patients

**Fig 2:**
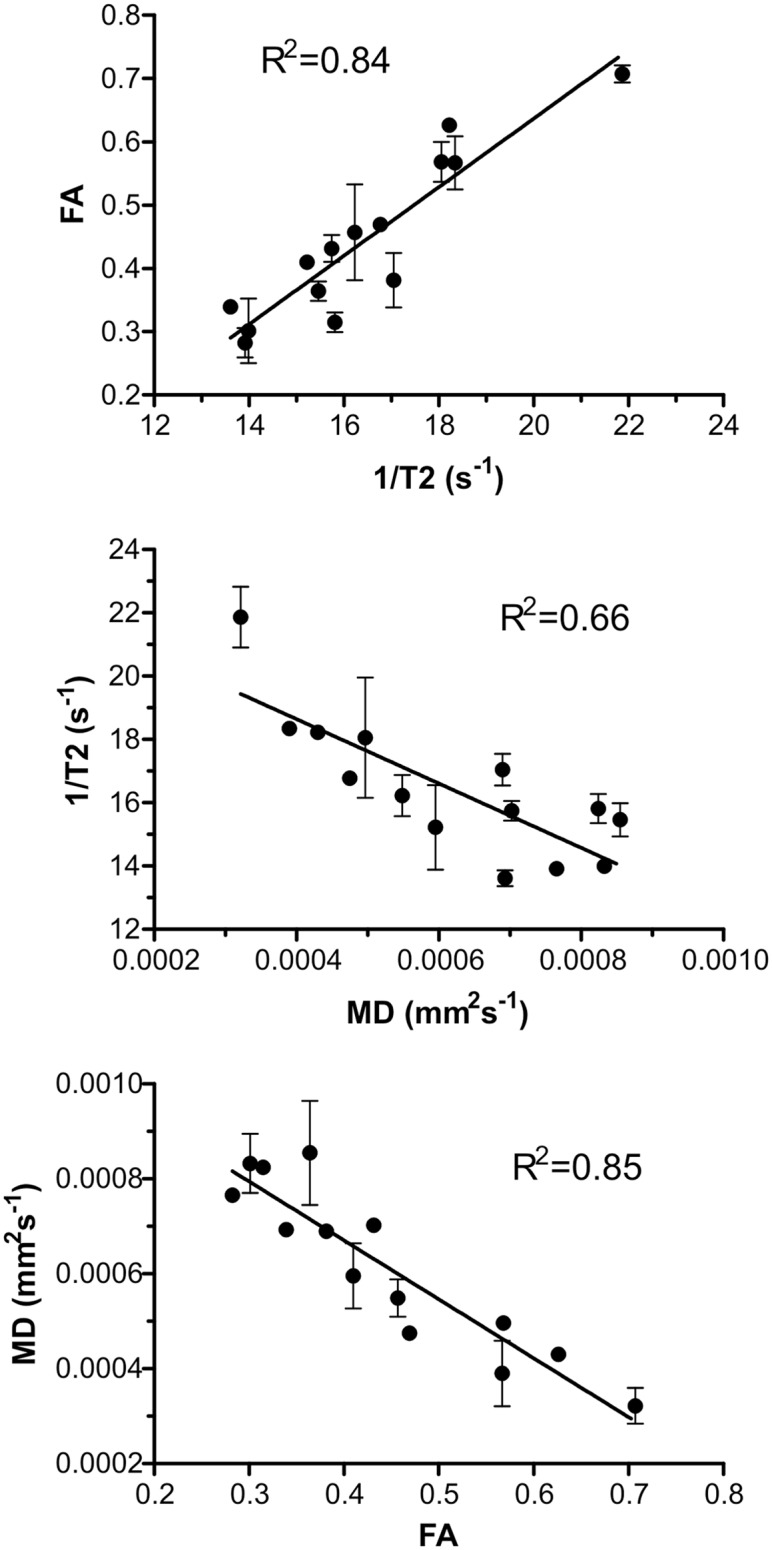
Correlation of relaxometry and diffusion parameters. The graphs describe the correlation between the relaxometry and diffusion data obtained from the pallidum of HD patients with corresponding standard deviations (SD). Top: correlation between RR (1/T2) and FA, middle: correlation between RR (1/T2) and MD, bottom: correlation between FA and MD.

No significant difference in RR, FA or MD was observed between the left and right pallidum. A positive linear relationship between RR and FA (R2 = 0.84, P<0.001, P<FDR) in the pallidum was found. A negative linear relationship was detected between RR and MD (R2 = 0.66, P<0.001, P<FDR) as well as between FA and MD (R2 = 0.85, P<0.001, P<FDR) in the pallidum. These data are displayed in Fig. 2. No significant relationship was found between FA or MD and RR in the pallidum of healthy control subjects.

**Fig 3:**
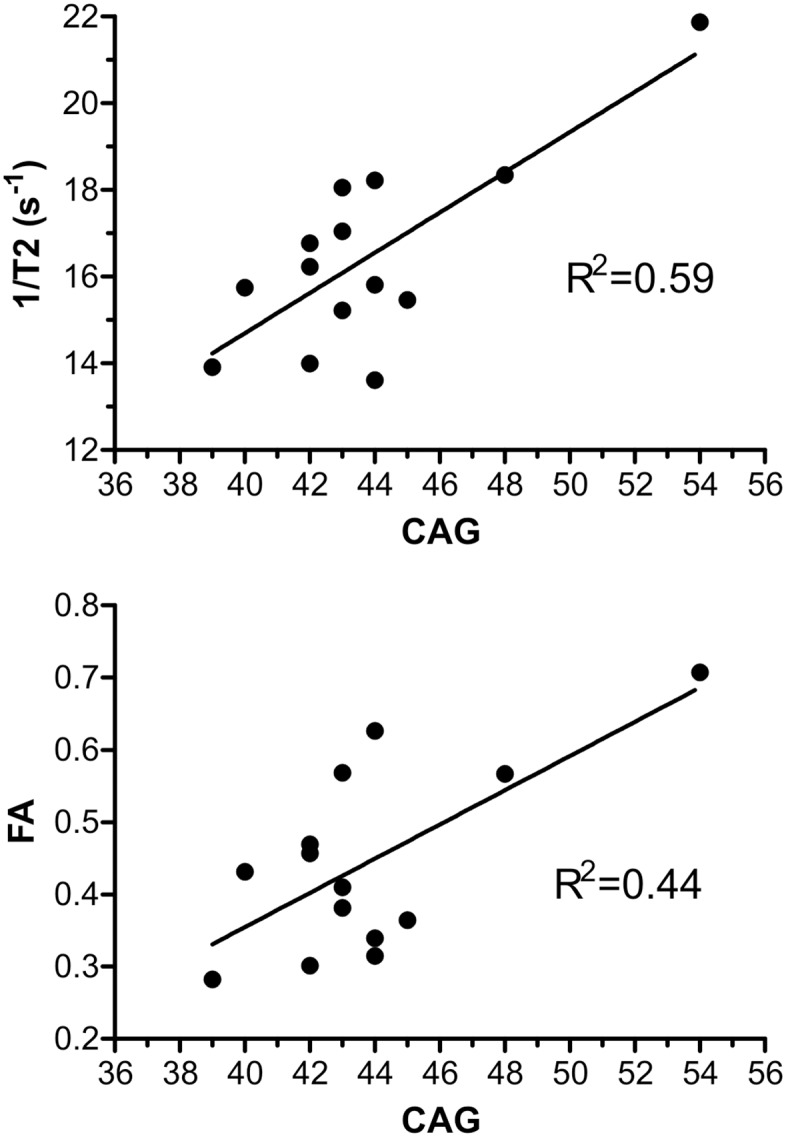
Correlation of MR data and number of CAG triplets. The graphs show the correlation between the MR data obtained from the pallidum of HD patients and the number of CAG triplet repeats. Top: correlation between RR (1/T2) and CAG, bottom: correlation between FA and CAG.

The average number of CAG triplet repeats was 45.5 ± 3.5 (SD) with a range of 39–54. A positive linear relationship in the pallidum between the number of CAG triplet repeats and RR (R2 = 0.59, P<0.05, P<FDR) as well as between the number of CAG triplet repeats and FA (R2 = 0.44, P<0.05, P<FDR) was observed. These data are displayed in Fig. 3. After correction for multiple comparisons no significant correlation between the number of CAG triplet repeats and MD (R2 = 0.31, P<0.05, P>FDR) was noted. The average number of DBS was 381.6 ± 98.5 (SD) with a range of 203–573.5. A positive linear relationship between the DBS and RR (R2 = 0.45, P<0.05) as well as between the DBS and FA (R2 = 0.30, P<0.05 and no correlation between the DBS and MD (R2 = 0.14, P>0.05) was found.

The mean motor subscale UHDRS score was 25.0 ± 7.1 (SD) with a range of 9–80 and the mean TFC score was 6.4 ± 0.7 (SD) with a range of 1–13. The mean duration of HD was 4.5 years with a range of 2–12 years. No correlation was found between the duration of HD, the onset age of HD or the age of patients and RR, FA or MD in the pallidum (R2<0.30, P>0.05, P>FDR). Similarly, no correlation was found between the motor subscale of UHDRS or the TFC score and the MR parameters (R2<0.30, P>0.05, P>FDR).

### Comparison of results from HD patients and healthy controls

**Fig 4:**
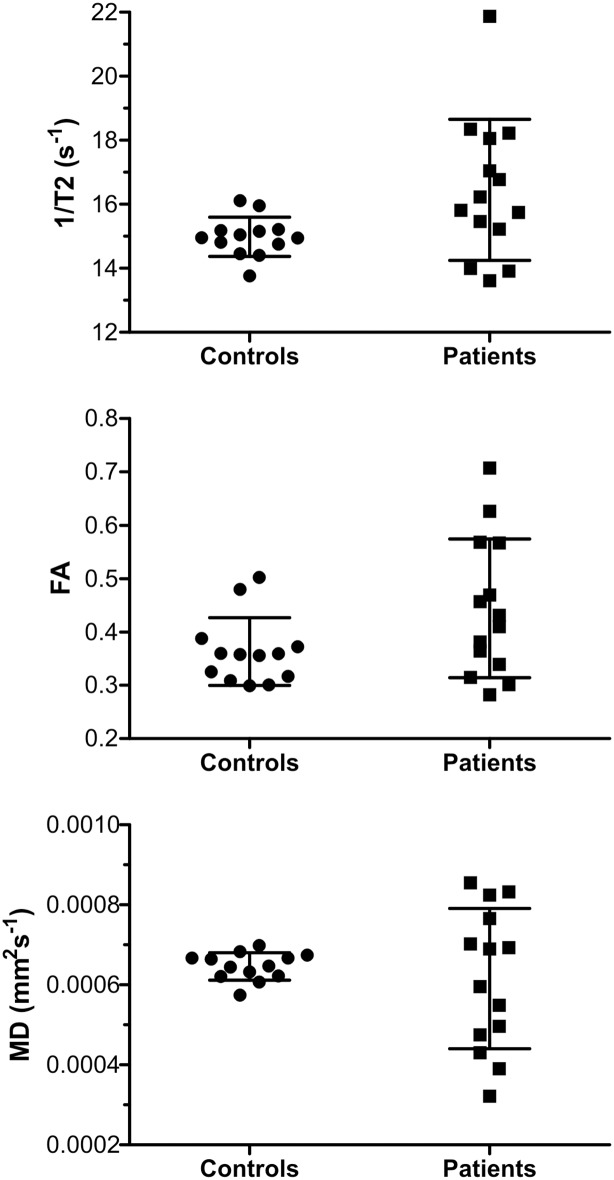
MR data in HD patients and healthy controls. The comparison of the MR data obtained from the pallidum of HD patients with the MR data obtained from the pallidum of healthy controls; the corresponding medians and average standard deviations are included. Top: comparison of RR (1/T2), middle: comparison of FA, bottom: comparison of MD.

No significant T2 relaxation time shortening (increase in RR) was found in the pallidum of HD patients compared to healthy controls (P>0.05). In addition to these relaxometry results, we also did not find any significant difference of FA and MD in the pallidum of HD patients compared to healthy controls (P>0.05). However, the RR, FA and MD data in patients were more scattered. These data are displayed in Fig. 4.

## Discussion

### Correlation of relaxometry, diffusion, genetic and clinical results from HD patients and control subjects

Our results demonstrate a strong relationship between relaxometry and diffusion parameters in the pallidum of HD patients. In reference to our recently published study [35], we suggest that an increased amount of metalloprotein-bound iron in this region can explain this relationship. The presence of metalloprotein-bound iron induces local susceptibility differences that enhance spin dephasing, thus decreasing T2 relaxation time (increasing RR). As DTI is T2-weighted, regions with greater metalloprotein-bound iron content have lower SNR. As FA strongly depends on SNR in low-SNR regions [35,41,42], the increased FA observed in the pallidum of HD subjects is likely due to decreased SNR. A possible explanation for the absence of a significant correlation between the relaxometry and diffusion parameters in healthy controls can be explained by the lower variance of metalloprotein-bound iron content in the pallidum of healthy subjects compared to HD patients.

On the other hand, it is known that DTI and T2 relaxometry measurements in the gray matter are not only dependent on metalloprotein-bound iron content [43], but are also affected by the extracellular space size, changes in the extracellular matrix composition, cell morphology and the presence of myelinated fibers [44]. Changes in tissue integrity, especially the selective loss of specific white matter tracts, have been suggested by some investigators to be responsible for the increase of FA observed in the putamen and pallidum of HD patients [11,12]. However, in field-dependant relaxometry studies the possible loss of tissue integrity was observed only in the putamen, and not in the pallidum of HD patients [29,33]. To measure T2 relaxation time we used a multiple echo sequence with a relatively short inter-echo spacing. Although this CPMG based sequence is very precise, due to the shorter diffusion time it may somewhat reduce the effect of metalloprotein-bound iron. Furthermore, the typical degenerative changes in HD, such as loss of neurons and gliosis, may increase T2 relaxation time and significantly contribute to the final T2 value. Therefore our absolute values of T2 relaxation times should be taken with precaution. On the other hand, in similar studies, the sequences with short echo times provided reliable data [45,46].

No observed correlation between RR, FA or MD in the pallidum of HD patients and clinical parameters may support the theory that an increased amount of metalloprotein-bound iron can obscure the possible relationship between the clinical results and the relaxometry or DTI data. This is supported by the fact that an increased amount of metalloprotein-bound iron is already present before the onset of HD symptoms [14]. No correlation between RR and clinical parameters is also consistent with the results of our previously published HD study [30].

The fact that RR in the pallidum of HD patients and the number of CAG triplet repeats are related has already been published [30]. Our new findings, the correlation between FA and the number of CAG triplet repeats, the correlation between FA and the compound variable of the number of CAG triplet repeats and the patient age—DBS, and the trend towards correlation between MD and the number of CAG triplet repeats, are consistent with our hypothesis about the metalloprotein-bound iron-mediated relationship between RR and DTI values.

The strong relationship between RR and FA or MD in our study is partly in disagreement with the results of a recently published multimodal imaging study in the BG of HD patients [14]. In this study only a trend towards correlation among increased RR*, increased FA and decreased MD was found. We do not compare the relaxometry results, since our RR data are not comparable to susceptibility influenced RR* data [47]. The explanation for the difference in the correlation between FA and MD can be found in the different methodologies used (different DTI sequences and slightly different postprocessing).

### Comparison of results from HD patients and healthy controls

In contrast to the recently published multimodal imaging study [14] we did not find a significant difference in FA in the pallidum of HD patients compared to the healthy controls; an initially significant difference in FA (and also RR) did not survive the restrictive correction for multiple comparisons. The explanation for this disagreement can again be found in the different methodologies used.

Our finding of no MD difference in the pallidum of HD patients compared to the healthy controls is in agreement with the results reported in the same multimodal imaging study [14] and one DTI study published earlier [13]. However, this finding is in disagreement with another DTI study on HD patients [12], where MD was reported to be increased. The different HD stage (TFC score) composition in the patient groups may explain the discrepancy between these results.

In conclusion, our results suggest that particular scalar parameters of DTI metrics in the gray matter may be influenced by metalloprotein-bound iron accumulation in the pallidum of HD patients. Consistent with this mechanism is our new finding of a correlation between FA data and the number of CAG triplet repeats. Furthermore, our results can explain the origin of the previously observed gray matter FA increases in neurodegenerative diseases [12] and to a large extent relate this finding to the presence of metalloprotein-bound iron. Future studies on a larger patient group, on animal models of HD and also in other diseases where iron accumulation is present should confirm to what extent the metalloprotein-bound iron concentration affects magnetic resonance measurements of gray matter diffusion parameters, especially in comparison to the above-mentioned tissue properties that are known to influence diffusion measurements.
